# “Nos violan y nos matan”: Violencias feminicidas y abandono institucional en la experiencia de niñas y adolescentes en centros de asistencia social en Jalisco, México

**DOI:** 10.18294/sc.2025.5334

**Published:** 2025-03-31

**Authors:** Gabriela Sánchez López, Rosa Elena Sandoval Zapata, Michelle Mir Trejo, Ana Cristina Skinfield Vértiz, María de los Ángeles Téllez Flores, Mónica Michelle Rojas Medina

**Affiliations:** 1 Doctora en Antropología Social. Profesora-investigadora, Instituto Tecnológico y de Estudios Superiores de Occidente, Universidad Jesuita de Guadalajara, Guadalajara, México. gabriela.sanchez@iteso.mx Universidad Jesuita de Guadalajara Instituto Tecnológico y de Estudios Superiores de Occidente Universidad Jesuita de Guadalajara Guadalajara Mexico gabriela.sanchez@iteso.mx; 2 Doctorante en Ciencias Sociales. Investigadora asociada y profesora, Instituto Tecnológico y de Estudios Superiores de Occidente, Universidad Jesuita de Guadalajara, Guadalajara, México. rosa.zapata0405@alumnos.udg.mx Universidad Jesuita de Guadalajara Instituto Tecnológico y de Estudios Superiores de Occidente Universidad Jesuita de Guadalajara Guadalajara Mexico rosa.zapata0405@alumnos.udg.mx; 3 Estudiante, Licenciatura en Psicología, Instituto Tecnológico y de Estudios Superiores de Occidente, Universidad Jesuita de Guadalajara, Guadalajara, México. michelle.mir@iteso.mx Universidad Jesuita de Guadalajara Instituto Tecnológico y de Estudios Superiores de Occidente Universidad Jesuita de Guadalajara Guadalajara Mexico michelle.mir@iteso.mx; 4 Estudiante, Licenciatura en Psicología, Instituto Tecnológico y de Estudios Superiores de Occidente, Universidad Jesuita de Guadalajara, Guadalajara, México. cristina.skinfield@iteso.mx Universidad Jesuita de Guadalajara Instituto Tecnológico y de Estudios Superiores de Occidente Universidad Jesuita de Guadalajara Guadalajara Mexico cristina.skinfield@iteso.mx; 5 Estudiante, Licenciatura en Psicología, Instituto Tecnológico y de Estudios Superiores de Occidente, Universidad Jesuita de Guadalajara, Guadalajara, México. maria.tellez@iteso.mx Universidad Jesuita de Guadalajara Instituto Tecnológico y de Estudios Superiores de Occidente Universidad Jesuita de Guadalajara Guadalajara Mexico maria.tellez@iteso.mx; 6 Estudiante, Licenciatura en Psicología, Instituto Tecnológico y de Estudios Superiores de Occidente, Universidad Jesuita de Guadalajara, Guadalajara, México. monica.rojas@iteso.mx Universidad Jesuita de Guadalajara Instituto Tecnológico y de Estudios Superiores de Occidente Universidad Jesuita de Guadalajara Guadalajara Mexico monica.rojas@iteso.mx

**Keywords:** Violencia, Feminicidio, Sistemas de Protección Infantil, Niñas, Adolescentes, México, Violence, Femicide, Child Protection Systems, Girls, Adolescents, Mexico

## Abstract

Este artículo examina el continuum de violencias feminicidas y su vínculo con los sistemas de protección en México, desde la perspectiva de niñas y adolescentes. A partir de un estudio cualitativo realizado entre 2022 y 2024 en dos centros de asistencia social, uno público y otro privado, se analizan las experiencias de 31 niñas y adolescentes que han atravesado este ciclo de violencias. Mediante entrevistas en profundidad, mapas corporales y observación etnográfica, se evidencia que las violencias arraigadas en sus comunidades de origen no cesan con su ingreso a los centros de asistencia social, sino que se transforman en nuevas formas de control y violencia institucional. A través de sus relatos, reconocemos una continuidad de violencias que configuran un entramado de despojo y deshumanización, en el que la institucionalización no representa un refugio, sino una extensión del mismo sistema de opresión. Los hallazgos muestran que los centros de asistencia social no son una ruptura con las violencias previas, sino parte del mismo continuum de control, exclusión y precarización de la vida, consolidándose dentro del entramado de impunidad regulado por el Estado.

## Abstract (en)

This article examines the continuum of feminicidal violence and its connection to protection systems in Mexico from the perspective of girls and adolescents. Based on a qualitative study conducted between 2022 and 2024 in two social care centers - one public and one private - it analyzes the experiences of 31 girls and adolescents who have gone through this cycle of violence. Through in-depth interviews, body mapping, and ethnographic observation, the study reveals that the violence rooted in their communities of origin does not cease upon entering the social care centers; instead, it is transformed into new forms of institutional control and violence. Their narratives reveal a continuity of violence that constitutes a web of dispossession and dehumanization, in which institutionalization does not represent a refuge but rather an extension of the same system of oppression. The findings show that social care centers do not break the cycle of previous violence, but rather form part of the same continuum of control, exclusion, and the precarization of life, becoming embedded within the broader framework of impunity upheld by the state.

## INTRODUCCIÓN

El feminicidio como categoría jurídica pertenece al ámbito del derecho penal y define el asesinato de mujeres por razones de género, estableciendo criterios específicos para su tipificación y sanción^1^. En contraste, proponemos las violencias feminicidas como continuum, es decir, como una categoría teórico-analítica que permite examinar las múltiples formas de violencia estructural y simbólica que configuran el contexto en el que estos crímenes ocurren^2^. Aunque operan en campos distintos, ambas perspectivas se interconectan al evidenciar la sistematicidad de las violencias contra las mujeres, desde su naturalización en la vida cotidiana hasta su expresión más extrema en el feminicidio.

Como señala Aguirre^1^, la tipificación del feminicidio en el derecho penal fue un proceso progresivo que tuvo como referencia el concepto de femicidio introducido por académicas como Jill Radford y Diana Russell en la década de 1970. Posteriormente, feministas en América Latina, como Marcela Lagarde, ampliaron su alcance para evidenciar no solo los asesinatos de mujeres, sino también las múltiples violencias estructurales y cotidianas que las afectan, resaltando sus implicaciones políticas y sociales^2^. Lagarde añadió la noción de impunidad y la construcción social detrás de estas muertes, enfatizando que los feminicidios son crímenes de odio enmarcados en relaciones de poder desiguales, con el perpetrador actuando desde una posición de supremacía^3^.

Sin embargo, se trata de un concepto teórico en disputa debido a la complejidad del fenómeno. Por ejemplo, Rita Segato^4^ va a distinguir a los feminicidios de los “crímenes de odio o pasionales” con la intención de separarlos de lo íntimo y del acontecimiento, para describir que la violencia contra las mujeres opera de forma sistemática y estructural. En ese sentido las violencias feminicidas ofrecen un marco de análisis más amplio para comprender los procesos que posibilitan el feminicidio, evidenciando que la violencia contra las mujeres no se limita a la privación de la vida, sino que operan de manera sistemática a través de una cadena de agresiones y mecanismos de control. Desde esta perspectiva, las violencias feminicidas no solo deben entenderse como un acto extremo, sino como parte de un fenómeno más amplio que abarca múltiples ámbitos de sociabilidad como la escuela, la familia, la pareja, el trabajo y la comunidad, en los que se perpetúan la desigualdad y el abuso, donde la responsabilidad del Estado es crucial. Esta noción permite reconocer cómo distintas formas de violencia -física, sexual, psicológica, económica e institucional-, incluidas la tortura, el secuestro, la desaparición y el desplazamiento forzado, están interconectadas en una continuidad de agresiones que se sostienen en el tiempo, atravesando la vida de las mujeres^2^^,^^5^^,^^6^^,^^7^^,^^8^.

Así, mientras la categoría jurídica del feminicidio busca nombrar y sancionar la expresión más extrema de esta violencia, el concepto de violencias feminicidas nos permite interrogar no solo los crímenes consumados, sino también las estructuras que los posibilitan y perpetúan, ampliándose a otras violencias de género que les anteceden.

Si las violencias feminicidas son la manifestación más extrema del continuum de violencia contra las mujeres, su existencia solo puede comprenderse dentro de un marco de necropolítica de género. Desde este marco interpretativo, la muerte, la precarización y la subordinación de los cuerpos feminizados no son excepciones, sino mecanismos fundamentales de un orden político y social^5^. La necropolítica fue formulada inicialmente por Achille Mbembe, el concepto describe la administración sistemática de la muerte como forma de gobierno, determinando qué vidas son protegidas y cuáles son desechables^9^. En este contexto, la necropolítica de género^5^ amplía esta perspectiva al revelar cómo los cuerpos de las mujeres y otras identidades feminizadas no solo son objeto de violencia letal, sino también de una lógica de disciplinamiento, explotación y sometimiento que opera a través del control sobre sus vidas, sexualidades y posibilidades de existencia^5^.

Este régimen de muerte no se sostiene solo a través de actores privados o criminales, sino que el Estado juega un papel central en su reproducción^10^. Desde esta perspectiva, podemos analizar cómo a través de la omisión, la impunidad y la normalización de la violencia, el Estado no solo permite, sino que en muchos casos legitima y administra la exposición sistemática de las mujeres a la muerte^10^. Esto se refleja en la falta de acceso a la justicia, de mecanismos de seguimiento y los procesos de la revictimización institucional^5^. Como señala Sagot^5^, la necropolítica de género se inscribe en un orden social patriarcal que legitima la violencia y dominación de las mujeres^5^. Así, las violencias feminicidas no solo son el resultado de acciones individuales, sino el producto de un sistema necropolítico, colonial y patriarcal que decide qué cuerpos pueden ser descartados sin consecuencias y bajo qué lógicas opera su exterminio^10^, dando cuenta del orden social de dominación que legitima, reproduce de forma sistemática estas violencias. 

### Tendencias y continuidad de las violencias feminicidas en México y Jalisco: afectaciones en niñas y adolescentes

Para abordar de manera empírica la dimensión de las violencias feminicidas en México y, particularmente, en Jalisco recurriremos a la información estadística disponible. Sin embargo, reconocemos que estos datos no representan la realidad del panorama de violencias. Al menos en el caso de Jalisco, el Instituto de Información Estadística y Geografía (IIEG) señala que la cifra negra de violencia, es decir, los datos que no son reportados, asciende al 91,6%, por lo que existe una clara subrepresentación estadística de las violencias^11^.

Pese a ello, los datos presentados por las instituciones son alarmantes, cuando hablamos de la persistencia de las violencias nos estamos refiriendo a la tendencia en aumento que han mostrado las defunciones de mujeres. Por ejemplo, entre 1985 y 2019 se registró que 63,324 mujeres en México habían sido asesinadas, de esa cifra, más de la mitad ocurrió entre 2007 y 2019. Tan solo en 2020 se registraron 1.932 mujeres asesinadas, de los cuales 489 fueron tipificados como feminicidios^12^. Los datos indican una ligera reducción en 2022 pero, rápidamente la tendencia volvió a aumentar^13^, siendo así que, para noviembre de 2024 los feminicidios reportados en el país fueron 733^14^. En ese sentido, resulta clara e irrefutable la persistencia y el incremento de las violencias contra las mujeres pero, al mismo tiempo, demuestra la lucha por visibilizar, nombrar y denunciar las violencias. 

La lectura de los datos en clave generacional nos muestra que, más de la mitad de las mujeres asesinadas en 2019 eran jóvenes, tenían entre 15 y 34 años, y en su mayoría fueron atacadas en la vía pública^12^. En el caso de Jalisco, entre enero y agosto de 2023 se reportaron 46 niñas y adolescentes (menores de 17 años) asesinadas. Durante el mismo periodo, pero en 2024, la cifra ascendió a 54^14^. Estos datos convierten a Jalisco en uno de los estados con mayor incidencia de feminicidios contra niñas y adolescentes solo por debajo del Estado de México y Veracruz. A nivel municipal, Guadalajara, Zapopan y Tlajomulco de Zúñiga figuran entre las localidades con mayor concentración de feminicidios en el país^15^. 

La información estadística presentada solo da cuenta del acto letal de las violencias, sin embargo, podemos aseverar que las experiencias de violencia comenzaron en edades muy tempranas. Los datos señalan un incremento en la frecuencia e intensidad según la edad, y muestran un aumento de la probabilidad de ser víctimas de feminicidio entre los cinco y nueve años, y luego, con la entrada a la adolescencia, entre los 15 y 17 años^16^. La Encuesta Nacional sobre la Dinámica de las Relaciones en los Hogares^17^ confirma esta tendencia en Jalisco, donde el 71,9% de las mujeres de 15 años y más reportaron haber sufrido algún tipo de violencia a lo largo de su vida, y de forma particular el 53,7% reportó que se trató de violencia sexual, con lo que identificamos el carácter central de este tipo de agresiones en el continuum de violencias feminicidas. 

Las cifras analizadas confirman que estas violencias no solo persisten, sino que afectan de manera desproporcionada a niñas y adolescentes. Ante este panorama, emerge la figura de los centros de asistencia social (CAS), espacios concebidos como lugares de resguardo para niñas y adolescentes en situación de violencia. No obstante, su funcionamiento plantea interrogantes sobre las lógicas de cuidado y control que operan en su interior y su capacidad real para garantizar la restitución de derechos. En una investigación previa, Sánchez^18^ analizó los motivos por los cuales las niñas y los niños ingresan al sistema de protección, destacando factores como el abandono, malos tratos y la violencia intrafamiliar. Sin embargo, estos análisis no daban cuenta de las violencias feminicidas ni de su impacto diferenciado en niñas y adolescentes. 

### Centros de asistencia social: institucionalización, precarización y continuidad de las violencias

La Ley General de Niñas, Niños y Adolescentes de 2014 estableció los CAS, (también conocidos como viviendas temporales o residencias alternativas), como espacios de acogida para niñas, niños y adolescentes en situación de vulnerabilidad o desamparo^19^. En los últimos años, la proliferación de CAS privados ha seguido una tendencia global en la que los Estados han delegado el cuidado de las infancias y adolescencias a actores privados, reduciendo su participación directa en estos servicios. Este proceso no solo refleja un cambio en la gestión del bienestar social, sino que también evidencia un debilitamiento del Estado, al transferir responsabilidades fundamentales a entidades privadas y reducir su rol como garante de derechos^20^. En México, este fenómeno ha resultado en una notable diferencia en la distribución de los centros: según la Comisión Nacional de los Derechos Humanos^21^, en 2018 se contabilizaron 877 CAS en el país, de los cuales 119 eran públicos y 562 privados. Esta disparidad no solo refleja la expansión del modelo privado, sino que también exige indagar quiénes llegan a estos espacios, qué violencias han atravesado antes de su ingreso y cómo estas continúan dentro de los CAS.

De acuerdo con el informe de la Comisión Nacional de los Derechos Humanos^21^, los CAS públicos en México, se concentran sobre todo en Jalisco y la Ciudad de México, y se caracterizan por su infraestructura para atender desde pequeños grupos hasta más de 200 personas. Cuentan con servicios médicos en el 97,1% de los casos, financiamiento principalmente estatal, y regulación y supervisión periódica. Además, deben contar con personal especializado en salud, trabajo social y psicología para garantizar un cuidado integral. Pueden contar con programas de educación formal e informal.

Los CAS privados están sujetos a la misma normativa que los públicos, aunque su supervisión y regulación son limitadas. La calidad de los servicios varía según los recursos disponibles y la gestión interna, lo que da lugar al hacinamiento, la falta de higiene e insumos básicos. Son gestionados por organizaciones civiles y religiosas, y predominan enfoques asistencialistas y disciplinarios en los que niñas y adolescentes enfrentan trabajo doméstico no remunerado y restricciones de movilidad como forma de control. A pesar de que estos centros deben garantizar acceso a derechos como la salud y la educación, persisten desigualdades en su provisión^21^^,^^22^. 

En Jalisco, varios CAS privados operan como centros de rehabilitación de adicciones, acogiendo a niñeces y adolescencias en extrema vulnerabilidad, sin distinguir entre quienes requieren atención por consumo problemático de sustancias y quienes han sido institucionalizados por otras razones. La falta de regulación, supervisión y mecanismos de denuncia, junto con la ausencia de protocolos adecuados que atiendan las necesidades específicas de niñas, niños y adolescentes, agravan la precariedad en estos espacios^21^.

Diversos estudios indican que la pandemia profundizó las problemáticas preexistentes en los CAS, en un contexto donde el Estado mexicano ya enfrentaba una crisis institucional de cuidados y de derechos humanos, dificultando la garantía de vida, integridad y desarrollo de quienes residen en estos espacios^23^^,^^24^^,^^25^. 

Estas condiciones, particularmente manifiestas en los CAS privados, pueden analizarse a la luz de la distinción que plantean Janet Finn y Lynn Nybell^20^ entre niñas, niños y adolescentes “en riesgo” y “riesgosos”, una clasificación que no es neutral, sino que refuerza desigualdades estructurales. Esta perspectiva permite cuestionar cómo ciertas poblaciones son atendidas bajo lógicas de protección, mientras que otras son sujetas a medidas de control, disciplinamiento o castigo. 

En diálogo con lo anterior, diferentes organismos han señalado que existe un uso desmedido de la institucionalización, priorizando el confinamiento sobre la restitución de derechos e integración comunitaria^19^^,^^20^^,^^21^^,^^26^. Así, se perpetúan las mismas lógicas de exclusión y castigo que Finn y Nybell^20^ identifican en el tratamiento de juventudes consideradas problemáticas. Este modelo de atención responde a discursos políticos, económicos y culturales que refuerzan la exclusión y la patologización de ciertas niñas y adolescentes, encubriendo las condiciones estructurales que determinan sus vidas en el capitalismo tardío^20^. Es frente a este panorama que nos preguntamos sobre las experiencias de violencia y de institucionalización de las niñas y adolescentes en los CAS públicos y privados. 

En este artículo exploramos cómo las niñas y adolescentes que residen en estos espacios perciben y procesan las violencias que enfrentan en un entorno que las desvaloriza e ignora. Analizamos cómo las políticas de muerte afectan a esta población, manifestándose en un continuum de violencias feminicidas que inician con la violencia sexual, y que se perpetúa en un contexto de impunidad sistemática. Desde la perspectiva de Segato^27^, esta realidad configura una pedagogía de la crueldad, en la que la violencia se internaliza como una constante inevitable y normalizada. Las narrativas provienen de niñas y adolescentes que han residido de forma temporal en CAS en Jalisco entre 2022 y 2024.

## PROCESO METODOLÓGICO: ESTRATEGIAS DE RECOLECCIÓN Y ANÁLISIS

### Participantes y contexto de estudio

El objetivo de este artículo es describir la crisis de los sistemas de protección de niñas y adolescentes en Jalisco frente al contexto de violencias feminicidas que atraviesa el Estado. Para ello dialogamos de manera continua desde septiembre de 2022 hasta junio de 2024, con 31 niñas y adolescentes. Al inicio de la investigación, 13 participantes tenían entre siete y doce años, y 18 eran mayores de trece años. Todas ellas vivían de manera temporal en dos CAS dependientes de los Sistemas Municipales de Protección de Niñas, Niños y Adolescentes, uno de ellos era público y otro privado. La primera autora realizó el trabajo de campo en el CAS público, mientras que en el CAS privado se incorporó la segunda autora y el resto del equipo. En total, participaron 12 niñas y adolescentes que residían en el centro público, mientras 19 permanecían en el privado. 

En ambos casos las niñas y adolescentes eran ingresadas a los CAS como una medida de protección, ya sea del Estado o de sus familias, con el objetivo de resguardarlas de agresiones sufridas en entornos familiares y comunitarios. El criterio de selección se basó en el interés manifestado por las propias niñas y adolescentes en participar, tomando en cuenta que todas habían atravesado experiencias de violencia y adversidad antes de su institucionalización. Así, su participación en el estudio fue voluntaria.

### Técnicas de recolección: escucha y observación en los CAS

Para comprender tanto el funcionamiento del sistema de protección, como la manera en que las violencias operan en la vida de las niñas y adolescentes, desde su propia experiencia, realizamos un estudio cualitativo basado en técnicas conversacionales y observación etnográfica.

A lo largo de la investigación, realizamos observaciones etnográficas que implicaron una presencia continua y el diálogo con las niñas y adolescentes en distintos momentos de su vida cotidiana, tanto dentro como fuera de los CAS. Estas observaciones fueron registradas en diarios de campo. Dentro de los CAS, la observación incluyó una participación constante en actividades diarias y la interacción en espacios de convivencia, terapias grupales y áreas recreativas. Esto permitió documentar las dinámicas internas, las normas de funcionamiento y las relaciones entre las niñas, adolescentes y el personal de los centros. Con relación a las técnicas conversacionales realizamos entrevistas en profundidad y conversaciones grupales en torno a mapas corporales. 

Además, realizamos 18 entrevistas individuales con adolescentes de entre 13 y 17 años. Cada entrevista tuvo una duración aproximada de 60 a 90 minutos y se llevó a cabo dentro de los CAS, en espacios previamente designados para garantizar privacidad y comodidad. Aunque partimos de una guía semiestructurada, la modalidad de la entrevista fue flexible para permitir que las adolescentes expresaran sus experiencias a su propio ritmo.

Finalmente, para reconocer las experiencias compartidas de violencia y generar una narrativa colectiva sobre éstas, realizamos conversaciones grupales con las 31 participantes en torno a la elaboración de mapas corporales. En total, se llevaron a cabo nueve sesiones, cada una con una duración de 120 a 180 minutos, en espacios comunes dentro de los CAS y con grupos de entre tres y cinco participantes.

Cada sesión inició con la elaboración del mapa corporal y continuó con una conversación grupal. La actividad consistió en dibujar la silueta de un cuerpo en cartulinas grandes, sobre la cual cada grupo representó, mediante dibujos y palabras, las experiencias de violencia que identificaban. Posteriormente, crearon un personaje ficticio basado en las vivencias compartidas, narrando en tercera persona los impactos de la violencia en tres dimensiones: biográfica, corporal y territorial.

El uso de la ficción y la ludicidad permitió generar un entorno seguro para hablar sobre las violencias sin exponer directamente las experiencias personales de cada participante^28^. En total, se elaboraron nueve mapas corporales que dieron forma a la misma cantidad de personajes ficticios inspirados en las experiencias reales de las niñas y adolescentes, con los que se elaboró, además, un relato colectivo.

Cabe señalar que la selección de técnicas cualitativas se realizó considerando la edad de las participantes. Mientras que con las niñas de entre siete y doce años solo se trabajó a partir de conversaciones grupales en torno a los mapas corporales, con las adolescentes de entre trece y diecisiete años se hicieron, además de mapas corporales, entrevistas en profundidad, permitiendo así captar tanto las dimensiones colectivas como las individuales de sus experiencias.

### Técnicas de análisis: entre categorías predefinidas y emergentes

Todas las entrevistas y conversaciones grupales fueron grabadas, con autorización por escrito de las participantes, y transcritas para su análisis. Aplicamos un enfoque híbrido de análisis de contenido, combinando una categorización deductiva basada en literatura previa sobre violencia, tomando como referencia el informe de UNICEF México *Panorama estadístico de la violencia contra niñas, niños y adolescentes en México*^29^, con una inductiva que permitió identificar dimensiones emergentes en los relatos.

Inicialmente, estructuramos una matriz analítica en dos dimensiones: 1) los espacios donde se perpetúa la violencia (comunitaria, en el hogar, institucional, escolar y de pareja) y los sujetos involucrados; y 2) sus expresiones (violencia armada, crónica, de género, emocional, física, negligencia, económica, sexual y estética). Sin embargo, al analizar los relatos, observamos que algunas de estas violencias coincidían con las categorías establecidas^29^, mientras que otras no eran reconocidas dentro de los marcos previos. En particular, las violencias feminicidas no estaban explícitamente contempladas, lo que nos llevó a una categorización inductiva para identificar cómo se expresaban en las narrativas de las niñas y adolescentes.

Las categorías previas de violencia^29^ no bastaban porque, no reflejaban la severidad de las violencias feminicidas que afectan de manera específica a niñas y adolescentes. En nuestro análisis, identificamos que estas violencias no solo implicaban agresiones individuales, sino que formaban parte de un entramado estructural de impunidad y control territorial que era sumatorio. Justificamos las nuevas categorías a partir de la recurrencia de estos patrones en los relatos, en los que emergieron formas de violencia como la descartabilidad, el feminicidio, la violencia física con intención letal hacia ellas u otros cuerpos feminizados, y la desaparición forzada, que no estaban contempladas en los marcos previos.

El enfoque híbrido nos permitió captar la complejidad y diversidad de las violencias narradas por las participantes, evidenciando cómo estas se entrecruzan en un continuum de violencias feminicidas.

### Consideraciones éticas

Para la participación de las niñas y adolescentes se obtuvo el consentimiento informado de cada una y los permisos necesarios de las personas adultas a su cargo, ya fueran representantes legales del Estado o familiares, por lo que se cumplió con los protocolos de consentimiento informado previamente acordados con la Comisión de Ética de la universidad y el Sistema de Protección de Niñas, Niños y Adolescentes. Esto incluyó el consentimiento verbal y la provisión de una hoja de información de contacto de la coordinadora de la investigación. Por último, para proteger la privacidad de las niñas y adolescentes, se alteraron todos los nombres de personas y barrios, así como otros datos secundarios que pudieran conducir a su identificación. 

Todo el proyecto de investigación se apegó de forma rigurosa y cuidadora a los lineamientos establecidos por el Comité de Ética del Instituto Tecnológico y de Estudios Superiores de Occidente (ITESO), Universidad Jesuita de Guadalajara (dictamen 0040CEI08-2022) y el equipo del Sistema Municipal de Protección Integral de Niñas, Niños y Adolescentes de Jalisco, quienes aprobaron el proyecto. 

## RESULTADOS

### Continuidades de violencia y exclusión: experiencias de niñas y adolescentes que residían en centros de asistencia social

Al presentar los hallazgos empíricos más significativos, somos conscientes de la crudeza de las narraciones y, aunque hemos omitido los detalles para evitar lo que Bourgois^30^ denomina “pornografía de la violencia”, su severidad sigue siendo innegable. Nuestra intención no es ni sobreexponer ni atenuar la gravedad de estas experiencias, sino dar cuenta de la magnitud del problema y las condiciones estructurales que lo sostienen. Además, el proceso de visibilizar la gravedad de la problemática responde a la agenda propuesta por las niñas y adolescentes, quienes demandaban ejercicios de escucha, sensibilidad compartida y reconocimiento de las realidades vividas. 

Para comprender la profundidad de estas violencias, analizamos primero las agresiones que las niñas y adolescentes enfrentaron en sus comunidades antes de su ingreso al CAS, evidenciando su progresión en un continuum que denominamos violencias feminicidas. Posteriormente, exploramos las experiencias dentro de los CAS, donde estas violencias no cesan, sino que adoptan nuevas formas que tensionan y desafían las redes de cuidado familiar, comunitario e institucional.

### El control del cuerpo y el territorio: violencias entrelazadas

Los ejercicios de diálogo colectivo sobre las violencias nos permitieron reconocer la primera de las continuidades en las experiencias, nos referimos al cuerpo/territorio. Mediante la creación colectiva mapas corporales, las participantes nombraron formas cotidianas de violencia en sus comunidades, hablaron de violencia delictiva, desapariciones forzadas y violencia sexual:

“*Por donde viven* [los personajes creados a partir de nuestras experiencias] *queman las casas y las saquean. Hay un horario para salir y, si sales fuera de ese horario, te matan. Hay mucha droga en las casas y en la calle. Es peligroso salir a jugar, ir a un mandado o andar con los amigos.* [Las mujeres] *tienen miedo de que las secuestren y de que las violen. No están seguras. Pero nadie hace nada; la policía tampoco las cuida de los peligros de la calle, y por eso se tienen que cuidar ellas mismas*”. (Relato colectivo de los mapas corporales, 4 de marzo de 2023)

La reflexión en colectivo de las violencias permitió reconocer prácticas comunes en todas las colonias en las que vivían antes de entrar al CAS. Todas coincidían en ser colonias de alta vulnerabilidad y marginalidad, ubicadas en las periferias sur y oriente de la zona metropolitana de la ciudad. Sus relatos daban cuenta de una concatenación de las violencias tejidas en la trama de lo cotidiano:

“*Pues ahí, diario se ve de robos, se ve de asaltos, de droga; se ve de violaciones. Diario se escuchan disparos; siempre se encuentran* [cuerpos] *embolsados en pedacitos*”. (Tania, 16 años, 7 de febrero de 2023)

La lectura social que las niñas y adolescentes hacen sobre sus barrios presenta un continuum en el que, por un lado, resulta inoperante analizar las violencias de manera taxonómica pues en sus relatos aparecen completamente imbricadas. La negligencia; las prácticas perjudiciales; el abuso físico, sexual y emocional se narran como sobrepuestas y constantes en sus vidas. Además, con mucha claridad señalaron que las violencias en sus barrios y familias se presentan de forma diferenciada por cuestiones de género. La invitación a reflexionar colectivamente sobre el cuerpo permitió reconocer la continuidad entre los conflictos y la búsqueda del control de los territorios, un dominio que se extiende también al control de los cuerpos. Cuando se le preguntó a María, de 14 años, cómo era el lugar donde vivía, respondió:

“*Bueno, después de las 10 ya no puedes salir porque es cuando comienzan a recoger a las niñas o a la gente* [...] *Las venden, se las llevan o las matan*”. (María, 14 años, 7 de febrero de 2023)

El relato de María coincidió con las observaciones de otras participantes, quienes describieron escenarios marcados por la presencia constante de mujeres capturadas y posteriormente abandonadas o arrojadas a la calle. A partir de estos testimonios, es posible interpretar que se trata del fenómeno de desaparición intermitente, analizado por Borzacchiello^7^, en el que adolescentes, principalmente de entre 14 y 16 años, son sustraídas durante un intervalo que oscila entre 72 horas y dos semanas, para luego ser devueltas a sus hogares o, en algunos casos, no regresar. Los relatos de las niñas y adolescentes en los CAS dan cuenta de dinámicas semejantes. Una de las participantes narró que estuvo desaparecida por dos días después de ser sustraída de un punto de venta de drogas -también llamado “paro o punto”- poco después de la intervención de la Guardia Nacional para desmantelarlo, lo que generó conflictos entre los grupos que gobiernan el territorio. La joven fue sometida a diferentes abusos, físicos y sexuales, antes de ser abandonada en la vía pública. Otra participante de 17 años, residente del mismo barrio relató lo siguiente:

“*Sí, de hecho, unos días antes mataron a una muchacha que nosotras conocíamos por allí. Luego, a la vuelta del punto, detuvieron a un señor por venta de blancas; tenía a varias muchachas ahí en casas. Así son las cosas*”. (Cindy, 17 años, 2 de febrero de 2023)

Nuevamente, en estos escenarios, las violencias se configuran como un continuum. Las adolescentes son privadas de su libertad y sometidas a violencia física y emocional, para luego ser forzadas a la explotación laboral o sexual. Según los relatos de las niñas y adolescentes, las desapariciones intermitentes no se presentan de manera aislada, la expresión “*así son las cosas*” evidencia la cotidianidad y persistencia de estas prácticas a lo largo del tiempo. Esto sugiere que no se trata de eventos excepcionales, sino de mecanismos sistemáticos de control de los cuerpos y territorios, cuyo carácter recurrente y sostenido suele quedar en la impunidad. La percepción de esta impunidad fue ampliamente compartida en la discusión colectiva de los mapas corporales. En ese espacio, las niñas y adolescentes expresaron lo siguiente:

“*En el barrio donde vive Ana* [el personaje que hemos creado a partir de nuestras experiencias], *perseguían a las muchachas y las llevaban a, pues, lo que Dios quisiera; nadie sabía lo que pasaba con ellas. Después de unos días, las encontraban, y era de su suerte si las encontraban vivas, o las encontraban muertas, o si las violaban y las dejaban vivas. Era lo que se veía ahí*”. (Relato colectivo de los mapas corporales, 4 de marzo de 2023)

El secuestro y la desaparición de niñas y adolescentes se inscriben como una forma extrema de objetivación de sus cuerpos y como un método de control territorial. Esta dinámica de violencia no solo despoja a las jóvenes de su libertad, sino que también refuerza el dominio sobre los territorios, utilizando sus cuerpos como recursos en una economía política de la violencia. En este contexto, Fernanda, de 14 años, relató que en esos territorios las reglas son claras:

“*Las muchachas de ahí no se pueden tocar sin el permiso de alguien del lugar. Siempre estábamos como vigilados por los del paro, por los del punto; que son los que controlan el área, los que mandan ahí*”. (Fernanda, 14 años, 2 de febrero 2023)

En palabras de Luisa, de 16 años: “*Me utilizaban muchísimo los hombres cuando estaba drogada, como su juguete*” (7 de febrero de 2023). La incapacidad de consentir en estos contextos es aprovechada para someter y violentar sexualmente a las adolescentes. A pesar de que muchas de ellas identifican el abuso con claridad, las condiciones en las que ocurren estas agresiones generan dinámicas de justificación o autoinculpación:

“*Entonces yo estaba muy alcoholizada. A lo mejor yo me culpo por eso también, por haber estado así. De hecho, ni siquiera me acuerdo exactamente de lo que pasó, ni siquiera sentí nada. Me bloqueé*”. (Jade, 17 años, 7 de febrero de 2023)

Este control sobre los cuerpos de niñas y adolescentes, ejercido a través del secuestro, la desaparición y la explotación sexual, no solo refuerza el poder territorial, sino que también despoja a las comunidades de su autonomía relativa. Al imponer sus reglas, estos grupos fuerzan a las niñas, adolescentes y sus familias a ajustarse a dinámicas de sometimiento que restringen su vida cotidiana y profundizan la erosión de su autonomía.

### Violencias feminicidas: continuidad, impunidad y transmisión generacional

Tal como anticipamos en el apartado metodológico, ante este panorama optamos por emplear una categoría emergente que nos permitió comprender con mayor precisión el fenómeno que estábamos acompañando y analizando en diálogo con las niñas y adolescentes. Este concepto, “violencias feminicidas”, nos permitió nombrar las violencias sistemáticas que se encadenan y se perpetúan.

Sin embargo, encontramos que estas violencias no se presentan de forma homogénea, ni sobre todos los cuerpos ni sobre todos los territorios. Más bien, se manifiestan de manera diferenciada según distintas categorías, como el género y la edad, como hemos venido desarrollando. El continuum de violencias adquiere características específicas y se intensifica en las experiencias de adolescentes que viven en zonas periféricas y en condiciones de precariedad, donde la sobrevivencia se vuelve central.

Las entrevistas y los diálogos colectivos con las niñas y adolescentes evidencian este panorama. Por ejemplo, de las 31 participantes, más de la mitad se identificó como sobrevivientes de violencia sexual; 22 relataron experiencias de violencia física severa, cuatro sobrevivieron a agresiones con intención letal, y tres se vieron forzadas a mudarse de ciudad o de barrio, debido a amenazas directas de muerte.

Los primeros abusos narrados ocurrieron entre los seis y nueve años de edad y continuaron durante la adolescencia. Sus agresores eran personas cercanas: familiares, vecinos y, posteriormente, parejas sentimentales o personas con quienes coincidieron en distintos espacios de socialización y a quienes consideraban amigos.

Además, estas violencias no solo afectaron sus cuerpos, sino también a las mujeres de su entorno inmediato: hermanas, madres, abuelas y amigas, quienes también han sido víctimas. Las violencias ejercidas contra las mujeres en sus círculos de cuidado más cercanos evidencian un patrón de continuidad generacional. Esta transmisión intergeneracional de la violencia se refleja en la experiencia de Fernanda, quien relata que su madre también fue violentada por sus parejas afectivas:

“*Mi padrastro le pegaba muy feo, la azotaba en la cama. Una vez llegó a lanzar un cuchillo y le cayó al lado a mi hermanito. He tenido muchos padrastros así. También mi mamá, ella no tuvo papá, y sus padrastros eran muy agresivos*”. (Fernanda, 14 años, 2 de febrero 2023)

El relato anterior evidencia otra de las continuidades de las violencias feminicidas: su perpetuidad a lo largo del tiempo. Estas violencias no solo se mantienen, sino que se transmiten de generación en generación. Según los resultados obtenidos, los agresores suelen ser varones dentro de los espacios íntimos e inmediatos de las niñas y adolescentes.

Por ejemplo, once participantes relataron que sus hermanos, padres o tíos intentaron matar a sus madres o madrastras; en dos casos, sus madres fueron asesinadas; y en cuatro experiencias, las niñas y adolescentes sufrieron la desaparición forzada de sus madres. En todos los casos, la violencia sigue impune.

Además de la impunidad que caracteriza estas violencias, resalta su dimensión generizada, ya que en la mayoría de los casos los agresores fueron varones. A su vez, estos se encontraban inmersos en otros procesos sistemáticos de violencia con características distintas, pero cuyas principales afectadas, directa o indirectamente, eran niñas, adolescentes y mujeres de su entorno cercano. Un ejemplo de esta dinámica se observa en el testimonio de una participante de 14 años, quien relató el allanamiento de su hogar mientras protegía a su hermana menor de un hombre armado que buscaba vengarse de su padre, amenazando la vida de ambas niñas. Además de este hecho, la misma participante describió otro episodio en el que logró sobrevivir a una desaparición intermitente, seguida de un ataque con intención letal, en el que otras niñas perdieron la vida en un contexto de trata y crimen organizado:

“*Mi hermano debía dinero, y pues llegaban a cobrarle a mi hermano, y mi hermano decía que no tenía. Y, pues, una vez yo iba saliendo de mi casa, y yo nada más recuerdo que me pusieron algo en la cara, una capucha. Me subieron y me empezaron a decir que esto iba por mi hermano, que todo esto lo estaba pagando por mi hermano, ¿no? Me durmieron y, ya en medio rato, desperté y amanecí en una bolsa de esas que usan los muertos* [...] *Se empezaron a escuchar gritos porque, aparte de mí, había más bolsas*”. (Mariana, 14 años, 7 de febrero de 2023)

Las experiencias y los datos compartidos hasta el momento evidencian la continuidad de las violencias experimentadas por niñas y adolescentes. Hemos identificado estas continuidades en tres dimensiones. La primera es la relación entre los cuerpos y los territorios: las descripciones de las niñas y adolescentes sobre sus barrios reflejan una organización social en la que la violencia se instrumentaliza para generar ganancias mediante el control y la mercantilización de los cuerpos.

La segunda continuidad radica en la concatenación de diferentes agresiones, donde múltiples formas de violencia se intersecan y refuerzan entre sí. Finalmente, la tercera dimensión es la persistencia de la violencia a lo largo del tiempo, configurándose como un fenómeno generacional.

Tal como mencionamos, la violencia feminicida emergió como una categoría analítica que nos permitió reconocer la articulación de distintas formas de violencia: física, sexual, emocional, el descuido y el trato negligente, todas ellas manifestadas simultáneamente. La convergencia de estas experiencias se hace evidente en el testimonio de Karen:

“*Mi papá siempre fue muy violento. Mis papás se separaron por eso, y entonces mi mamá me abandonó. Después me encontró y, cuando vio cómo estaba, me metió a un anexo. Seguía el problema con mi papá hasta que un día mi mamá me comentó que a mi papá lo estaban buscando para matarlo y… ese día, en la noche, me volvió a marcar y mi papá ya había fallecido; tenía un tiro en la cabeza*. […] *Con mi pareja fue muy difícil. Él creía que yo era de él y hacía lo que quería conmigo; llegó el punto en donde él me pegó bien feo. Yo pensaba que nunca iba a salir de ese infierno, que iba a durar toda la vida y que me iba a terminar matando; no le veía salida a eso. Yo, con tantito que pisara la calle, él corría detrás de mí y me metía de las greñas enfrente de la gente. Él era muy violento. Recuerdo que traía vestidos y él me los quitaba en la calle, dejándome encuerada, sin nada. Me quemaba mis tenis; me quitaba todo. Entonces, llegó el punto en donde yo estaba embarazada, pero no me di cuenta, y él, de tan fuerte que me golpeó, me provocó un aborto*”. (Karen, 16 años, 7 de febrero de 2023)

En este breve fragmento de su vida, Karen relata haber presenciado violencia en su ámbito familiar, abandono, consumo de sustancias, violencia letal con el asesinato de su padre, así como violencia emocional, sexual y física ejercida por su pareja. Su testimonio expresa con profunda claridad lo que entendemos por violencia feminicida, particularmente, cuando afirma: “*Yo pensaba que nunca iba a salir de ese infierno, que iba a durar toda la vida y que me iba a terminar matando; no le veía salida a eso*”.

Se trata, entonces, de una violencia que atraviesa todos los ámbitos de la vida, que se manifiesta de múltiples formas y cuyo desenlace pareciera ser, inevitablemente, la muerte. Es por esta razón que proponemos el término violencias feminicidas, para referirnos a un continuum de violencia que, aunque no siempre culmina en la muerte, se encuentra en una constante tendencia hacia ella y, además, se reproduce de manera generacional.

### Invisibilidad y abandono en los sistemas de protección: los CAS y la violencia institucional

Las experiencias narradas no solo desafían las definiciones convencionales sobre violencias infanto-adolescentes, sino que también evidencian las complejidades que enfrentan los sistemas de cuidados comunitarios, familiares e institucionales. En este análisis, nos hemos centrado en las violencias y vulneraciones vinculadas al género y la edad. En este sentido, las niñas y adolescentes han sido violentadas en espacios que, en principio, deberían garantizar protección y cuidado. La mayoría ha sufrido violencia en sus entornos familiares y de pareja. Frente a esta realidad, los CAS se presentan como espacios de resguardo para niñas y adolescentes.

Sin embargo, la impunidad estructural genera una paradoja en la que la estancia en estos centros es percibida como un castigo, confinando a las niñas y adolescentes en lugar de a los agresores. Esta percepción se manifiesta en el testimonio de Kendra, una adolescente de 15 años, quien quedó embarazada a los 13 como consecuencia de una violación:

“*Él está libre, está afuera. Por eso yo no me puedo ir; él es un riesgo y tienen miedo de que me vea con el niño. A mí me había dicho mi licenciado que lo iban a llevar a la penal, pero hace dos años ya de eso y no ha pasado nada* […] *Yo digo que él fue el que hizo el delito y no yo, ¿para qué estoy aquí en un proceso, encerrada, y él disfrutando de la vida? Tampoco sé si se lo está haciendo a otras niñas lo que me hizo a mí*”. (Kendra, 15 años, 2 de febrero de 2023)

De este modo, los CAS son percibidos como espacios de encierro forzado que, además, no siempre cumplen con sus objetivos de resguardo y protección. La violencia no cesa en estos centros; por el contrario, se reproduce como un continuum, alimentada por la informalidad y la precariedad de las instituciones. Encontramos que, en muchos casos, los CAS se convierten en espacios que vulneran nuevamente a las niñas y adolescentes, en lugar de garantizar su seguridad.

Esta realidad es expresada con claridad en el testimonio de Sofía, de 17 años:

“*La primera vez que yo llego a un anexo fue hace unos meses. A ese anexo yo llego porque ya no tenía dónde vivir* [...] *El padrino de ahí todavía se seguía drogando y, en chinguiza, nos hizo a mí y a otras compañeras madrinas, pero nos seguíamos drogando con él, porque quería que nos acostáramos con él. Entonces, ese fue mi primer proceso*”. (Sofía, 17 años, 7 de febrero de 2023)

Sin embargo, pese a este panorama, el fenómeno es aún más complejo. Los CAS privados han comenzado a operar de manera emergente e informal como refugios a los que las familias llevan a sus hijas para protegerlas de las amenazas y agresiones con intención letal en sus barrios. En un contexto de extrema violencia e impunidad, estos centros han sido concebidos como espacios de resguardo frente a la falta de respuestas del Estado.

De manera que, esta condición de protección ha sido instrumentalizada por algunos centros como mecanismo de control, bajo el argumento de que las jóvenes “*no pueden salir porque las matan*”. Como resultado, muchas adolescentes prolongan su estancia en los CAS, donde terminan sosteniendo las necesidades de cuidado del centro a cambio de techo y comida.

Esta percepción de los CAS como un refugio indispensable se expresa en el testimonio de Fernanda, de 14 años:

“*Estamos protegidos porque afuera están matando a muchas muchachas. eso es lo que nos platicaban otra vez, que estaban matando muchas muchachas, que le diéramos gracias a Dios porque estábamos protegidas de que el día de mañana no fuéramos nosotras*”. (Fernanda, 14 años, 2 de febrero de 2023).

Los relatos de las niñas y adolescentes sobre las violencias feminicidas evidencian la normalización de la muerte y la descartabilidad, configurando un escenario en el que la vida misma se vuelve precaria y prescindible. Esta precarización instala una tensión constante entre vivir en el exterior, expuestas a violencias letales, o ingresar a los CAS, donde la violencia institucional se reproduce bajo nuevas formas.

## DISCUSIÓN

Este estudio revela que las niñas y adolescentes se encuentran atrapadas en un continuum de violencias feminicidas, en el que las agresiones no son eventos aislados, sino que se acumulan y escalan en contextos de impunidad y explotación^2^^,^^31^^,^^32^^,^^33^^,^^7^^,^^40^^,^^35^. Nuestros hallazgos coinciden con Fragoso^32^ y Borzacchiello^7^ en la necesidad de analizar estas violencias desde una perspectiva geopolítica, pues no pueden entenderse únicamente como tendencias delictivas, sino como fenómenos estructurales vinculados al territorio y las relaciones de poder que lo configuran. En este sentido, nuestro estudio también muestra que la violencia sexual y las desapariciones intermitentes dentro de sus comunidades refuerzan la conexión entre el control sobre los cuerpos y los territorios, un patrón que también han documentado Ravelo^34^ y Borzacchiello^7^. Ravelo ha analizado cómo la violencia sexual opera como un mecanismo de sometimiento dentro de sistemas de control socioeconómico y criminal, mientras que Borzacchiello^7^ ha examinado la desaparición intermitente en relación con estas mismas dinámicas de violencia estructural. Nuestros hallazgos se inscriben en esta línea de análisis, evidenciando que las violencias feminicidas no pueden entenderse de manera aislada, sino como parte de un entramado de dominación que entrelaza las violencias con estructuras económicas y criminales que perpetúan la impunidad con la tolerancia del Estado.

Nuestros hallazgos coinciden con los de Fragoso y Fernández^35^ en cuanto a que la violencia sexual contra niñas y adolescentes es una de las agresiones más recurrentes dentro del continuum de violencias feminicidas, que no ocurre de manera aislada, sino en conjunción con otras formas de violencia. Sin embargo, mientras su estudio se centra en las manifestaciones extremas de esta violencia en el feminicidio infantil, nuestros resultados muestran que muchas niñas y adolescentes sobreviven a estas dinámicas en condiciones de vulnerabilidad sostenida. Siguiendo su análisis, encontramos que la degradación de lo femenino o en sus términos, la *basurización* de los cuerpos -elementos claves en los feminicidios infantiles- también están presentes en los entornos y experiencias de quienes han sido sometidas a un continuum de violencias, aunque no hayan culminado en muerte. Esto refuerza la necesidad de comprender la violencia sexual como parte de un proyecto de deshumanización o necropolítica de género^5^.

Además, esta violencia no se limita al espacio público, sino que se articula con agresiones en ámbitos privados, que se reproducen y normalizan dentro de redes de cercanía y confianza. En este sentido, nuestros hallazgos coinciden con los de García^36^, Contreras, Bott, Guedes y Dartnall^37^ y Niño^38^ en que los agresores suelen pertenecer al círculo más próximo -familiares, vecinos, parejas sentimentales- lo que no solo perpetúa el abuso, sino que también restringe la posibilidad de que las víctimas identifiquen espacios seguros o construyan redes de apoyo.

Las violencias feminicidas, en especial la violencia sexual contra niñas y adolescentes, funcionan como mecanismos políticos y ejemplarizantes que refuerzan estructuras de poder, opresión y descartabilidad. Estas estrategias deliberadas imponen un mensaje de dominación sobre las mujeres^13^ y de acción-actuación para los hombres^20^. Esto se enmarca en lo que Segato^27^ plantea sobre las pedagogías de la crueldad, que no solo disciplinan y someten, sino que también normalizan la desvalorización y descartabilidad de ciertos cuerpos. Nuestros hallazgos refuerzan esta perspectiva, mostrando cómo la impunidad, desde edades tempranas, inscribe en niñas y adolescentes la convicción de que sus vidas son prescindibles.

Esta desvalorización no solo se manifiesta en la violencia comunitaria y familiar, sino que también se reproduce en las instituciones encargadas de la protección. Nuestro estudio se suma a las investigaciones que han evidenciado la precarización en los CAS, donde la institucionalización, lejos de proteger, expone a niñas y adolescentes al control, la explotación y la desubjetivación^22^^,^^25^^,^^21^.

La falta de vigilancia y acompañamiento del Estado, como señala Ruiz^39^, configura estos espacios como “espacios de riesgo”, donde los cuidados de sus residentes quedan supeditados a criterios de gestión institucional que priorizan la homogenización y el control. Esta lógica no responde a las necesidades de las niñas y adolescentes, sino a las de los adultos que operan los CAS, reforzando dinámicas de despersonalización y exclusión. 

Nuestros hallazgos refuerzan esta perspectiva. En lugar de ofrecer cuidados, los CAS terminan reproduciendo la violencia, la invisibilidad, el abandono institucional y la explotación de su tiempo y energía en tareas de cuidado y mantenimiento del centro, sin reconocimiento ni retribución. Lejos de brindar alternativas reales para su desarrollo y autonomía, perpetúan su vulnerabilidad.

Por otro lado, hemos identificado un fenómeno poco documentado en otros estudios: el continuum de violencias ha llevado a que, paradójicamente, muchas familias vean en los CAS una alternativa de refugio para sus hijas. Ante la falta de seguridad en sus comunidades, los perciben como el único espacio donde pueden alejarlas -al menos temporalmente- de situaciones que amenazan sus vidas, aunque esto implique exponerlas a nuevas formas de violencia, precarización y control institucional. Esto no solo refuerza su vulnerabilidad, sino que las sitúa en una desventaja social estructural, marcada por el aislamiento y la ausencia de alternativas reales de autonomía.

Todos estos fenómenos se inscriben en una dinámica más amplia, donde la impunidad y la falta de respuestas institucionales perpetúan la desprotección de las víctimas, sometiéndolas a esquemas de mercantilización y control que refuerzan su exclusión y deshumanización tal como ha sido ampliamente documentado^32^^,^^35^^,^^33^^,^^40^^,^^7^^,^^34^.

## REFLEXIONES FINALES

A lo largo de esta investigación, identificamos dos conclusiones principales acerca de las experiencias de las niñas y adolescentes que participaron. La primera señala que el concepto de violencias feminicidas permite comprender el continuum de violencias que atraviesa la vida de las niñas y adolescentes, sin detenerse con el proceso de institucionalización. “*Nos violan y nos matan*” fue una expresión utilizada por las participantes para describir este continuum de violencias y la impunidad. Esta expresión enfatiza la relación entre la violencia sexual y la muerte, así como su vínculo con otras formas de opresión, como los secuestros, las desapariciones intermitentes, la violencia física extrema y las amenazas de muerte.

Los hallazgos del estudio permiten identificar tres connotaciones fundamentales para comprender las violencias feminicidas: 1) se dirigen específicamente a los cuerpos de mujeres; 2) se configuran como una concatenación de distintas formas de violencia que operan simultáneamente; y 3) constituyen un continuum persistente en el tiempo, con un carácter generacional que tiende a intensificarse, lo que agrava su letalidad al acumular múltiples experiencias previas de violencia ejercida contra ellas, sus hijas y cuidadoras. Los hallazgos de la investigación son congruentes con lo encontrado por otros estudios^4^^,^^41^^,^^42^^,^^43^ que coinciden en que la violencia contra las mujeres opera como una violencia instrumental y expresiva. Los cuerpos feminizados son utilizados como dispositivos de legitimación del poder dentro de una estructura patriarcal y jerárquica.

A lo largo del texto, desarrollamos cómo la continuidad de las violencias se articula a través del tiempo y los espacios. Las niñas y adolescentes describieron límites difusos entre el cuerpo y el territorio, así como entre generaciones, evidenciando la perpetuidad de las violencias feminicidas a lo largo del tiempo y su arraigo en los territorios y en todos los ámbitos de la vida cotidiana.

La segunda conclusión de nuestro estudio se relaciona con el análisis de los sistemas de protección del Estado. Encontramos que los Centros de Asistencia Social (CAS) enfrentan problemáticas altamente complejas. Si bien operan bajo una lógica de resguardo, en la práctica funcionan como espacios de contención de las violencias feminicidas presentes en los barrios y las familias; pero, al mismo tiempo, producen y reproducen otras formas de violencia a través del control, la exclusión, los cuidados negligentes y la precarización.

Ante el debilitamiento y la omisión del Estado, los CAS funcionan como dispositivos de legitimación y administración de la violencia sistemática, confinando y excluyendo a las niñas y adolescentes sin garantizar mecanismos efectivos de protección, justicia y reparación. El hecho de que sean vistos como la única alternativa refuerza el rol del Estado como gestor de las violencias y evidencia la ausencia de opciones reales de atención y contención, dejando a niñas y adolescentes en una situación de desprotección. Tal como ellas mismas lo expresan, el proceso de institucionalización es percibido como un castigo, lo que refuerza la sensación de que las violencias se encadenan y persisten, manifestándose bajo diferentes lógicas e intensidades, pero siempre como parte del mismo continuum.

En ese sentido, a partir de los datos recopilados, se concluye que los CAS operan bajo las lógicas de necropolítica de género, desempeñando una función burocrática, administrativa y de gestión de la muerte y el sufrimiento sobre los cuerpos y vidas de niñas y adolescentes^44^. Desde esta perspectiva, la violencia feminicida no solo se manifiesta en asesinatos y desapariciones, sino también en la administración diferencial de la vida: mientras algunas son asesinadas públicamente, secuestradas o violentadas, otras son invisibilizadas dentro de instituciones que, bajo la apariencia de protección, terminan confinándolas en entornos que perpetúan su vulnerabilidad.

Para finalizar, destacamos algunas líneas de investigación que requieren una mayor profundización. Tras el análisis realizado, reconocemos la necesidad de incorporar una perspectiva interseccional que articule las condiciones geográficas, socioeconómicas y generacionales. En este estudio, nos centramos en analizar la dimensión local del fenómeno, enfocándonos en los CAS en Jalisco, México. Sin embargo, nuestros hallazgos evidencian la importancia de ampliar la mirada para comprender la continuidad de las violencias feminicidas entre territorios. Queda pendiente un análisis que examine los flujos de desplazamiento de niñas, adolescentes y sus familias en un marco nacional e internacional de violencias, así como su relación con los sistemas de protección y cuidados estatales y globales.

Por otro lado, encontramos que las violencias se manifiestan de manera desigual y diferenciada según la intersección de estas condiciones. En particular, resaltamos la urgencia de analizar la violencia desde una perspectiva generacional, interseccional, relacional y de continuidad. Esto cobra especial relevancia ante la tendencia, tanto a nivel nacional como internacional, de clasificar las violencias de forma taxonómica. En el caso de las infancias y adolescencias, este enfoque fragmentado omite dimensiones cruciales como la descartabilidad, la violencia física con intención letal y la desaparición intermitente.

Investigaciones previas han advertido que las clasificaciones estatales tienden a homogeneizar la violencia bajo categorías generales, diluyendo o ignorando las formas en que afectan de manera diferenciada a niñas y adolescentes^18^. La ausencia de categorías adecuadas para nombrar estas violencias no solo contribuye a su invisibilización, sino que también obstaculiza su reconocimiento en las políticas de protección y justicia, perpetuando un sistema que encubre la desprotección bajo el discurso de la tutela estatal. Esta omisión impide comprender cómo estas violencias operan como un continuum de despojo y exterminio.

Tal como advierten Larraín *et al*.^45^, la violencia contra las niñas se diluye dentro de la categoría general de “violencia infantil”, la cual suele centrarse en niños varones, mientras que los estudios sobre violencia de género han privilegiado el análisis de mujeres adultas. Esta fragmentación en los datos impide una lectura integral del problema y refuerza la invisibilización de las violencias específicas que enfrentan las niñas y adolescentes. No obstante, según muestran las experiencias, es necesaria además una perspectiva relacional, que contemple a las niñas y adolescentes vinculadas con sus hijas, madres, tías y abuelas. Esta perspectiva permite observar con mayor profundidad la perpetuación de la violencia a través de diferentes generaciones, al mismo tiempo que visibiliza las redes de cuidado que operan en lo cotidiano y por fuera del Estado. 

Por ello, proponemos, para futuros estudios, un análisis de las violencias que articule las dimensiones territorial, interseccional, relacional y generacional, con el fin de dar cuenta tanto de sus continuidades como de sus especificidades.
